# Anthocyanin-Rich Butterfly Pea Petal Extract Loaded Double Pickering Emulsion Containing Nanocrystalline Cellulose: Physicochemical Properties, Stability, and Rheology

**DOI:** 10.3390/foods12224173

**Published:** 2023-11-19

**Authors:** Pankaj Koirala, Jiratthitikan Sriprablom, Thunnalin Winuprasith

**Affiliations:** Institute of Nutrition, Mahidol University, Nakhon Pathom 73070, Thailand; pankajkoirala50@gmail.com (P.K.); jiratthitikan.sri@mahidol.ac.th (J.S.)

**Keywords:** anthocyanin, nanocrystalline cellulose, double emulsion, rheology

## Abstract

Butterfly pea petal extract (BPE)-loaded water-in-oil-in-water (W/O/W) emulsions were fabricated using nanocrystalline cellulose (NCC) as a hydrophilic stabilizer and polyglycerol polyricinoleate (PGPR) as a hydrophobic emulsifier. The impact of different concentrations of NCC and PGPR in different phase proportions on the emulsion formation, rheology, and stability of an anthocyanin-loaded (pH ≈ 7.0) emulsion was investigated. The mean droplet size of the emulsions increased as the NCC concentration increased, while color intensity (greenness) decreased as the PGPR and NCC concentrations increased. A microscopic examination confirmed that the NCC nanoparticles stabilized the inner W_1_/O phase, whereas the excess concentration of non-adsorbing NCC nanoparticles was suspended in the continuous aqueous phase. The rheological results showed that robust emulsion networks were formed when the NCC concentration increased. A network structure between the droplets and the development of the NCC network during the continuous phase were attributed to a gel-like behavior. Over the course of seven days, the emulsions with a higher proportion of NCC remained stable, as in samples 3%P-%N, 5%P-2%N, and 5%P@1%N, the total anthocyanin content decreased from 89.83% to 76.49%, 89.40% to 79.65, and 86.63% to 71.40%, respectively. These findings have significant implications for the accurate formulation of particle-stabilized double emulsions for anthocyanin delivery with higher stability.

## 1. Introduction

Anthocyanin is well known for its bioactive and coloring properties as well as its promising pharmaceutical and industrial applications. The biological functions of anthocyanin are associated with a significant role in the modulation of oxidative stress and inflammation, perhaps delaying the onset of noncommunicable chronic diseases [1]. Typically, anthocyanin from the butterfly pea, *Clitoria ternatea*, is a potent inhibitor of nuclear NF-κB translocation and possesses a non-ROS suppression mechanism because of its high ternatin (polyacylated anthocyanin pigment) content [2]. The aqueous extract of butterfly pea petals lowers blood pressure, controls diabetes, and has a protective effect on human erythrocytes against hemolysis [3]. Further, the aqueous extracts of butterfly pea petals have demonstrated decreased levels of insulin resistance, hepatic malondialdehyde, free fatty acids, cholesterol, low-density lipoprotein, and plasma leptin [1]. The phytochemicals identified in the extract of butterfly pea, including flavanols (e.g., procyanidin A2, quercetin-3-rutinoside, epicatechin, myricetin, and kaempferol), phenols (syringic, gallic, *p*-coumaric, protocatechuic, ferulic, and caffeic acids), triterpenoids, and anthocyanins (e.g., ternatin), are responsible for the biological functions stated above [4,5]. The development of therapeutic and functional foods from BPEs has piqued the interest of the food industry. The direct application of the extract to products is limited due to the molecular instability of bioactive compounds in different environmental conditions, including temperature, light, pH, and oxygen. Additionally, anthocyanin has a low bioavailability when ingested directly because of the gastrointestinal pH, digestive enzyme activity, and the biotransformation of the microbiota [6,7]. Consequently, it is necessary to develop efficient techniques that are able to increase their stability and bioavailability.

The application of nanotechnology to entrap bioactive compounds like anthocyanin can be a viable alternative to overcome its chemical instability [8]. Double-emulsion-based approaches demonstrate the potential to successfully incorporate and deliver both hydrophilic and hydrophobic bioactive compounds. It is a liquid dispersion system where the droplets of liquid are entrapped by another liquid, producing double-layered liquid droplets. In such a system, the inner phase is covered by emulsifiers, which are further dispersed and covered by the outer layer of emulsifiers. Broadly, it is classified as water-in-oil-in-water (W/O/W) or oil-in-water-in-oil (O/W/O) double emulsions, regardless of the exact structure of the inner phase [9]. Double emulsions are an intricate multiphase system where both hydrophilic and hydrophobic interfaces are present [10]. The encapsulation of anthocyanin compounds in a double emulsion increases the stability of the emulsion while also protecting against different environmental stresses [10]. However, the existence of two interfaces necessitates the use of two emulsifiers to stabilize the inner and outer phases. This may lead to an excessive consumption of synthetic surfactant. Hence, double Pickering emulsions could be an alternative approach to forming stable emulsions, where the interface is stabilized by substituting solid particles from natural sources instead of a synthetic surfactant. In double Pickering emulsions, the adsorption of solid particles at the interface theoretically precludes both droplet coalescence and the interchange of emulsifiers across interfaces [11]. The novel concept of using nanocrystalline cellulose (NCC), also known as cellulose nanocrystals (CNC), as an emulsifier, on the other hand, is being researched extensively as a green, sustainable, and low-cost form of Pickering emulsifier. Rescignano et al. [12] fabricated double Pickering emulsions with poly lactic-co-glycolic acid nanoparticles and NCC. They found that NCC as an emulsion stabilizer for the formation of nanoparticles ensured the stabilization of the nanoparticles in aqueous dissolution.

Several studies have documented the preparation of emulsions integrated with NCCs [13,14]. To the best of our knowledge, however, the use of NCCs to stabilize Butterfly pea petal extract (BPE)-loaded double Pickering emulsion has not yet been studied. The objective of this work was to understand the influence of operational parameters such as emulsifier concentration, and the phase volume proportion on emulsion properties, rheology, microstructure, and encapsulation efficiency to successfully encapsulate BPEs in the inner phase of a water-in-oil-in-water emulsion. The effect of various operational parameters on the physiochemical properties (visual creaming, morphology, surface charge, and particle size distribution) and rheological properties was studied. Optimized double Pickering emulsions were subjected to a structure analysis by confocal laser scanning microscopy (CLSM) and an encapsulation efficiency analysis during storage.

## 2. Materials and Methods

### 2.1. Materials

Commercially available spray-dried nanocrystalline cellulose, extracted from Northern bleached softwood kraft pulp, was acquired from Cellulose Lab Company, Fredericton, NB, Canada. As per the supplier, the NCC specifications had dimensions of 100 to 260 nm, a ζ-potential of −40 mV, and a sulfur content of 0.8 wt%, which comes from the negatively charged sulfate half-ester (OSO^3−^) groups formed during the sulfuric acid hydrolysis process. Polyglycerol polyricinoleate (PGPR) was obtained from Sigma-Aldrich, Inc., St. Louis, MO, USA. Butterfly pea petal extracted by ultrasound-assisted extraction was obtained from NAP Biotec Co., Ltd., Nakorn Pathom, Thailand, where delphinidin was the major anthocyanin compound (Figure S1). Soybean oil was obtained from a local supermarket in Nakhon Pathom with no further purification. Additional chemical reagents, including potassium sorbate, were of analytical grade.

### 2.2. Double Pickering Emulsion Preparation

BPE-loaded water-in-oil-in-water (W_1_/O/W_2_) emulsions were formulated using a two-step emulsification process at room temperature (25 °C). The W_1_/O emulsions were initially formed by combining the aqueous and oil phases in a constant volume ratio of 3:7, where the aqueous phase contained 5% (*w*/*v* of the inner phase) BPEs in deionized water at pH 7, and the oil phase (O) contained polyglycerol polyricinoleate (PGPR) in soybean oil. The mixture obtained was homogenized in a homogenizer (HG-15A equipped with the stator dispersing tool HT1025, Daihan Scientific Co., Ltd., Wonju, Republic of Korea) for 3 min at 20,000 rpm, held for 10 min, and re-homogenized for 1 min at 10,000 rpm. Based on the preliminary results, the exterior aqueous phase (W_2_) with varying percentages of NCC was prepared. In the following step, the initial emulsion (W_1_/O) was blended with the W_2_ phase with a weight ratio of 2:8 or 3:7. A homogenizer was used to homogenize the mixture for 2 min at 15,000 rpm. For each formulation, 0.05% (*w*/*v*) potassium sorbate was added as an antimicrobial agent, and the experiment was carried out at least three times.

Different proportions of PGPR (3 and 5% *w/v* based on oil volume), NCC powder (1, 2, or 3% *w/v* based on final volume), and internal-to-external-phase ratios (2:8 and 3:7) were all tested experimentally (Table 1). The analysis was conducted after a 24 h storage at ambient temperature to achieve complete dispersion.

### 2.3. Particle Size and ζ-Potential Measurement

Particle size and the distribution of each emulsion were analyzed using a laser diffraction particle size analyzer (PSA 1190; Anton Paar GmbH, Graz, Austria). Distilled water was used to dilute all samples to avoid multiple scattering effects, and the refractive indices of distilled water and oil used for the calculation phases were set to 1.33 and 1.46, respectively.

A particle electrophoresis instrument (Zetasizer Nano ZS, Malvern Instruments, Worcestershire, UK) was employed to determine the electrical charge (ζ-potential) of the double emulsions. Distilled water was used to dilute the emulsion to avoid multiple scattering effects.

### 2.4. Visual Creaming Assessment

The freshly prepared emulsion was placed in a 20 × 60 mm cylindrical glass tube and sealed with a plastic cap, after which the sample was allowed to equilibrate for 24 h. After obtaining the reading on day 1, the tube was stored at ambient conditions for 14 days. Phase separation tests were performed on the emulsions at 1, 3, 5, 7, and 14 days to determine their stability. The following equation was used to calculate the creaming index (CI) as reported by Sriprablom et al. [15]:

(1) $$CI\%=\frac{H_{s}}{H_{t}}\times 100\;$$

where *H_s_* is the bottom layer height, and *H_t_* is the total emulsion height in the cylinder.

### 2.5. Color

A colorimeter (CR-20, Konica Minolta Sensing Americas, Inc., NJ, USA) was used to determine the color of the BPE-loaded double emulsion. A standard white plate was used to calibrate the colorimeter. The L* (lightness), a* (redness/greenness), and b* (yellowness/blueness) values were extracted.

### 2.6. Rheological Properties

The rheological properties (viscosity and flow behavior) of all emulsions were measured at 25 °C using a rheometer (HAAKE™ MARS™, 40 Rheometers, Thermo Fisher Scientific Inc., Waltham, MA, USA). The rheometer was outfitted with a cone and plate sensor (2° cone angle, 40 mm diameter, and 0.05 mm gap) made of titanium. All freshly prepared samples were stored for 24 h before analysis and then analyzed in triplicate.

A linear viscoelastic range (LVR) was determined by strain sweep (0.01–100%) tests at a fixed frequency of 10 rad/s. Subsequently, dynamic frequency sweep tests (from 0.1 to 100 rad/s) were performed with a constant strain of 0.5% within the linear region. The mechanical characteristic of the emulsions was measured by recording the storage modulus (G′) and the loss modulus (G″) as functions of frequency.

Steady-flow tests were performed by programming the cone of the measuring sensor to linearly increase the shear rate from 0.1 $s^{-1}$ to 300 $s^{-1}$ for 3 min followed by an immediate decrease from 300 $s^{-1}$ to 0.1 $s^{-1}$ for the next 3 min. All flow curves were fitted with the following power-law model:

(2) $$\sigma =k{\left(\gamma^{\circ }\right)}^{n}$$

where σ, γ^∘^, *k,* and *n* represent the shear stress (Pa), shear rate (s^−1^), consistency coefficient (Pa.s^n^), and flow behavior index, respectively [16,17].

### 2.7. Confocal Laser Scanning Microscopy (CLSM)

Confocal laser scanning microscopy was used to observe the microstructure of BPE-loaded double-emulsion droplets. For staining the oil droplets, aliquots of 0.01% (*w*/*v*) Nile red solution in methanol (15 μL) were added to samples (0.5 mL). An aliquot (15 µL) of the mixture was spread on a microscopic glass slide and carefully covered with a coverslip. The BPE-loaded double emulsions were examined using a 40× objective lens on an FV1000 CLSM (Olympus Corporation, Tokyo, Japan). To scan the images, the excitation wavelength was adjusted to 488 nm.

### 2.8. Encapsulation Efficiency (EE)

The encapsulation efficiency of the BPE-loaded double emulsions was determined using methods modified from Lin et al. [10]. The EE and encapsulation stability (ES) were determined by quantifying extract release from the inner phase into the outer phase after preparation and storage for 7 days. The fresh emulsions were stored in a serum bottle at room temperature after preparation. Before analysis, stored samples were diluted with distilled water and centrifuged (4000× *g* for 10 min). The aqueous phase of the subnatant was carefully recovered and filtered. The concentration of the extract was determined by obtaining the absorbance from a UV–vis spectrophotometer at a wavelength of 530 nm. EE and ES were calculated using the following equation:

(3) $$EE\left(\%\right)=\frac{C_{w1}\times X_{w1}-C_{i}\times \left(D+X_{w2}\right)}{C_{w1}\times X_{w1}}\times 100$$

(4) $$ES\left(\%\right)=\frac{C_{w1}\times X_{w1}-C_{s}\times \left(D+X_{w2}\right)}{C_{w1}\times X_{w1}}\times 100$$

where *C_W_*_1_ indicates the initial BPE concentration in the inner aqueous phase, *C_i_* is the BPE concentration in the outer aqueous phase right after the formation of a double emulsion, *C_s_* is the BPE concentration in the external aqueous phase after a period of storage, *X_W_*_1_ and *X_W_*_2_ are the mass fractions of the internal and external aqueous phases of the emulsion, and *D* is the dilution factor.

### 2.9. Statistical Analysis

Unless otherwise specified, all analyses were performed in triplicate. The results were expressed in terms of means  ±  standard deviation. A statistical analysis (one-way ANOVA) was performed using the SPSS version 16.0 Windows application (SPSS Inc., Chicago, IL, USA).

## 3. Results and Discussion

### 3.1. Characterization of Anthocyanin-Loaded Double Pickering Emulsions

The particle size, size distribution, surface potential, color, and creaming stability of BPE-loaded double emulsions with varying concentrations of polyglycerol polyricinoleate (PGPR) and nanocrystalline cellulose (NCC) were examined as well as variations in the phase volume proportion within the double emulsions to gain a thorough understanding of the properties and stability characteristics inherent in these emulsions.

#### 3.1.1. Particle Size and Distribution

**Figure 1:**
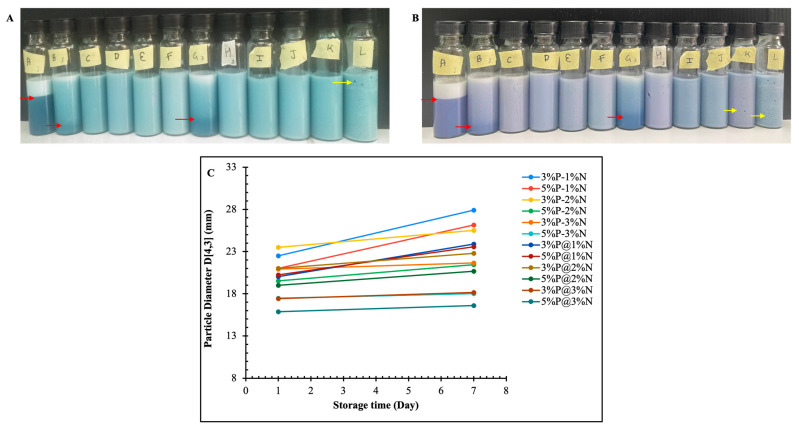
Creaming stability of BPE-loaded double Pickering emulsions after 1 day (**A**) and 7 days of storage (**B**). The particle diameter of BPE-loaded double Pickering emulsions stabilized by various PGPR and NCC concentrations and prepared with different phase volumetric ratios of W_1_/O in W_2_ (**C**). Red arrows denote the phase separation, while yellow arrows mark the formation of air bubbles.

The appearance of the freshly prepared and 7-day-storage emulsions are shown in Figure 1A,B, respectively. On day 1, the mean diameters of the particle size based on weighted volume, which is denoted as D [4,3], of the BPE-loaded double emulsion ranged from 15.9 ± 0.9 to 23.5 ± 1.1 μm, depending on the concentration of PGPR, NCC, and phase volume. In comparison, the increasing PGPR concentration and outer-phase proportions influenced the particle size and distribution. For double emulsions with 5% *w/v* PGPR, the particle size decreased with increasing concentrations of NCC, whereas the trend was irregular for 3% *w/v* PGPR with increasing concentrations of NCC. Figure 1C demonstrates the change in mean diameters of the particle size based on weighted volume at day 1 and after 7 days of storage, where there was a significant increase in the particle diameter after storage. Likewise, the mean diameters of the particle size based on weighted area, which is denoted as D [3,2], of the BPE-loaded double emulsion showed similar trends concerning the different concentrations of PGPR and NCC (Table 2). A narrow distribution of particle size was observed, with a shift to a lower particle size with the increasing NCC concentration (Figure S2). The findings in the current study are consistent with the results disclosed by Bai et al. [18], where it became apparent that the concentration of nanocrystalline cellulose in the oil–water emulsion had a significant effect on particle size. Bai et al. [18] noted that the mean droplet diameter decreased with the increasing concentration of NCC (from 0.05 to 0.75% *w*/*w*) and plateaued (~1 μm) when it was increased from 0.75 to 2% *w*/*w*. A lower NCC concentration restricts the stable emulsion formulation since it is insufficient to prevent coalescence. A higher NCC content results in a more stable emulsion since more negative charges are present and also increases the emulsion viscosity [14,19].

In the present findings, a decreased particle size was observed when the concentration of PGPR was increased from 3% to 5%. Irrespective of phase proportion and NCC, the mean particle size of the emulsions containing PGPR at 5% *w/v* was lower compared to the emulsion with 3% PGPR. However, the difference in particle size was not statistically significant except for compositions with 2% (*w*/*v*) NCC. The result aligns with research by Panagopoulou et al. [20], which kept the hydrophilic stabilizer constant and varied the concentration of PGPR from 1.6% to 3.2% *w*/*w* of double emulsion. The oil particle size decreased with the increasing PGPR content at a fixed bacterial cellulose concentration. With an increase in the concentration of PGPR from 2.5 to 4%, W_1_/O emulsions that are stable throughout coalescence were reported. This could be because, as a lipophilic emulsifier, PGPR saturates the inner water droplet surface by lowering the interfacial tension between the water and the oil, which inhibits destabilization [21]. Thus, it is crucial to determine the critical concentration of NCC and PGPR in double-emulsion formulations.

#### 3.1.2. ζ-Potential

**Figure 2:**
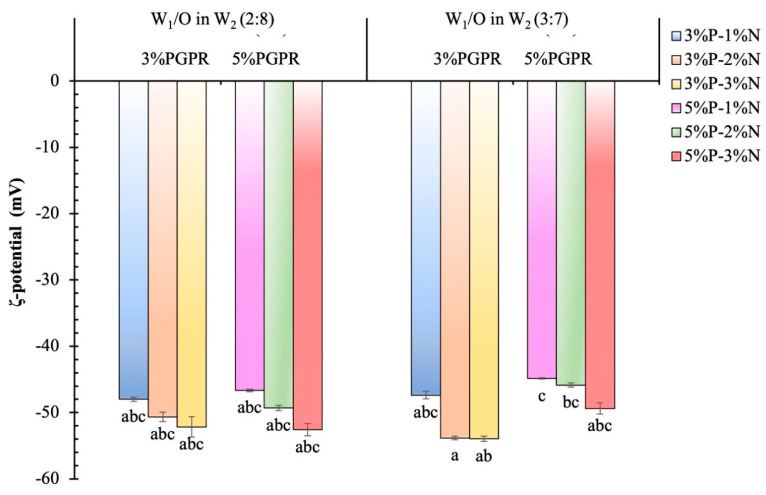
ζ-potential distributions of BPE-loaded double Pickering emulsions stabilized by various PGPR and NCC concentrations and prepared with different phase volumetric ratios of W_1_/O in W_2_. Different lowercase letters (a–c) indicate a significant (*p* ≤ 0.05) difference concerning different formulations.

As displayed in Figure 2, all W_1_/O/W_2_ emulsions had negative potential values, and the change in the ζ-potential of the BPE-loaded double emulsion with regard to the changing concentration of NCC ranged from −44.85 ± 0.1 mV to −53.95 ± 0.4 mV. The varying concentrations of PGPR have no significant contribution to the charge of the emulsion system. The absolute value of the ζ-potential was higher when the NCC concentration was increased. A sulfated NCC has a higher negative charge [22], which contributes to increasing the negativity of the charge at the interface. Additionally, the stability of the emulsion could be enhanced by the electrostatic repulsion driven by the higher negative charge, which could effectively avoid droplet collision and aggregation [23]. Due to an increase in NCC adsorption, an increase in NCC concentration could cause the monolayer to reorganize and the interface to become denser. Consequently, the close packing of NCC at the interfaces of the oil droplets could help to prevent the emulsion from coalescing during storage [24]. However, excessively high charges triggered excessive electrostatic repulsion, which prevented NCC from adhering to and aligning at the oil–water interface [25].

#### 3.1.3. Visual Appearance

The CIE (L*, a*, and b*) color parameters were measured for freshly prepared BPE-loaded W_1_/O/W_2_ to monitor color changes for all studied systems (Table 2). Concerning the pH adjustment to 7, it aims to increase the greenness of the emulsion. The a* (redness/greenness) parameter decreased with increasing concentrations of PGPR and NCC. In the case of b* (yellowness/blueness), the blueness value decreased with increasing NCC concentration, but the result was not significantly different concerning the formulation factors (PGPR, NCC, and the outer phase proportion). A likely reason for the decrease in greenness and blueness is that the increasing NCC concentration sufficiently incorporates the W_1_/O, while a low NCC concentration is unable to span the inner anthocyanin-containing phase. Similarly, the intervening oil phase, which acts as a barrier, was further stabilized with higher concentrations of PGPR and lower BPE diffusion to the outer phase. The color of anthocyanin is affected by the location of the anthocyanin in the emulsion system [26]. This result can be attributed to changes in the absorption spectra (diminished greenish color shift) of the anthocyanin when it is encapsulated in a double emulsion with increasing concentrations of PGPR [27]. Thus, appropriate concentrations of PGPR and NCC in relation to phase proportions during the stable emulsion formulation are crucial for protecting the color change in the emulsion system during storage.

#### 3.1.4. Visual Creaming

The visual creaming of BPE-loaded double emulsion is shown in pictures of the respective formulations in Figure 1A (for preparation day) and Figure 1B (after seven days of storage). The stability of an emulsion is also determined by the creaming index, where higher values indicate instability in the emulsion system. Only the double emulsions containing 1% NCC exhibited the creaming phenomenon after 1 day of storage, except for 5% (*w*/*v*) PGPR with an internal-to-external-phase ratio of 3:7. Samples 3%P-1%N, 5%P-1%N, and 3%P@1%N displayed creaming indexes of 75.6 ± 1.0, 15.6 ± 1.3, and 32.0 ± 2.1, respectively, which suggested a lower concentration of NCC was insufficient to stabilize the W_1_/O phase. No visual creaming was observed in the BPE-loaded double emulsion with a higher concentration of NCC. In an emulsion system, the viscosity of the emulsion is strongly dependent on the NCC concentration, as the three-dimensional network structure formation between the continuous phase and droplet interface helps to prevent aggregation and coalescence [28,29]. McClements [28] reported the potential effect of Stokes’ law, as it suggests a higher continuous phase viscosity could be attributed to preventing the creaming phenomenon.

Although the emulsion with a higher NCC concentration (3% *w*/*v*) stabilized the W_1_/O on storage, air bubbles were abundant. Saffarionpour [30] reported that a strong network formation between particles and increasing concentrations of NCC led to a more stable emulsion. Due to coupled electrostatic and steric repulsions between droplets containing NCC, NCC offers an excellent resistance against coalescence regardless of the low interfacial NCC coverage [19,31]. Moreover, the adjustment of the pH in the inner water phase could have contributed to the stability of this multiphase system. The pH of the inner aqueous phase was initially adjusted to pH 7, which resulted in the pH of all emulsions being around 6.8 (data not mentioned). This may also have contributed to the stability of the BPE-loaded double emulsion. As with the adjustment of pH, the interface charge can be changed, which also affects surface wettability, interfacial tension, and particle adsorption capacity [32]. Xiao et al. [33] utilized carboxymethyl starch nanoparticles to stabilize pH-responsive Pickering emulsions, where the emulsion was highly viscous and had excellent stability between pH 6.0 and 10.0. As mentioned previously, the higher concentration of NCC (3%) had a strong stability in emulsion, and air bubbles were abundant upon storage. The emulsion morphology was irregular, leading to core loss [34].

### 3.2. Rheological Properties

**Figure 3:**
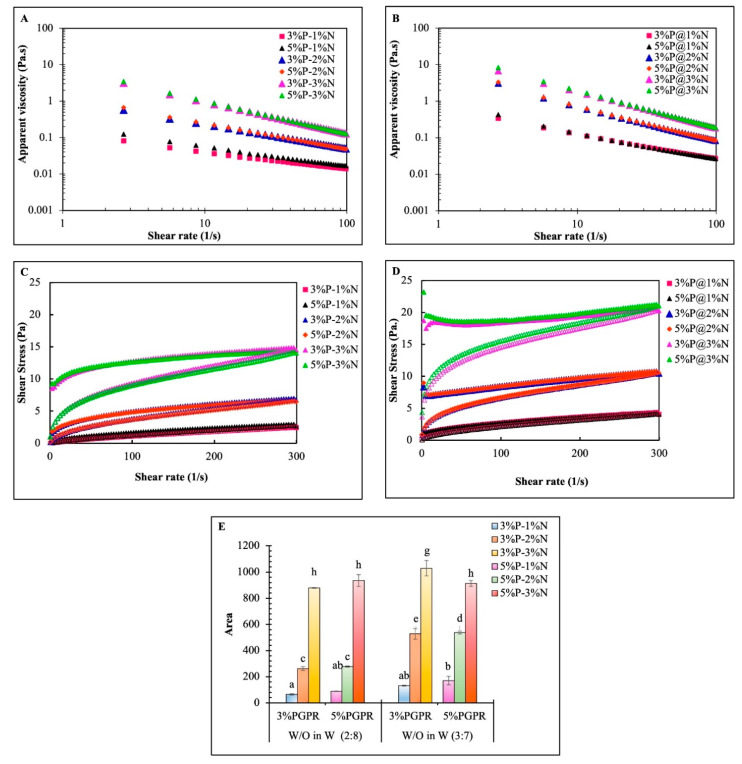
Steady flow behavior of BPE-loaded double Pickering emulsions stabilized by various PGPR and NCC concentrations and prepared with different phase volumetric ratios of W_1_/O in W_2_: apparent viscosity (Pa.s) vs. shear rate (1/s) (**A**,**B**), shear stress (Pa) vs. shear rate (1/s) (**C**,**D**), and hysteresis area (**E**). Different lowercase letters (a–e, g, h) indicate a significant (*p* ≤ 0.05) difference between the samples.

Regarding steady shear tests, the plots of apparent viscosity (*η*_a_) versus shear rate with different phase volumetric ratios of W_1_/O in W_2_ are depicted in Figure 3A,B, and shear stress versus shear rate graphs are in Figure 3C,D. With increasing shear rates, all emulsions exhibited a shear-thinning behavior. Furthermore, the double emulsion displayed a higher viscosity when the NCC content was increased, and the emulsion with 3% NCC at the phase volume (W_1_/O in W_2_) of 3:7 showed the highest viscosity. Due to strong interactions between NCC nanoparticles at higher concentrations, the resultant thickening effect leads to a higher viscosity [35]. Additionally, the nonadsorbing nanocellulose at high concentrations might cause flocculation through bridging or depletion mechanisms, which would increase viscosity at a low shear rate [36,37], resulting in retarding the phase separation during storage. As the shear rate increased, however, such interaction rapidly diminished as a result of deformation, and liquidlike properties became dominant. These phenomena suggested that all emulsions were susceptible to structural breakdown at high shear rates. In particular, the droplets had a high viscosity and moved as bulky aggregates at low shear rates. At high shear rates, however, the droplets were rapidly deformed and aligned in the shear flow direction, which reduced the resistance to flow [38,39]. Hence, all emulsions displayed a closer viscosity at a high shear rate (100 s^−1^) regardless of the amount of NCC.

All emulsions with the 5% PGPR discussed in this paper showed a slightly higher viscosity compared to the 3% PGPR (Figure 3A,B) due to the more compact structure and smaller size of oil droplets [20]. For shear stress versus shear rate, flow curves of all double emulsions exhibited a mainly time-dependent shear-thinning (thixotropic) yield-stress behavior in the shear rate range tested (Figure 3D,E). Quantitatively, the thixotropic properties were determined by measuring the area of the hysteresis loop (mPa s^−1^) at various concentrations of NCC and PGPR. The double emulsion with a composition of 3% PGPR and 3% NCC had the highest thixotropicity as it corresponded to a high hysteresis area. All suspensions displayed a typical thixotropic behavior, as shown in Figure 3C, which is typically associated with systems with flocculated particles or aligned nanocrystals. For formulations with a 1% and 2% NCC content, the size of the hysteresis loop was rather small, indicating that the NCC had a weak thixotropic tendency, and the microstructure damaged by shearing could be restored shortly [40,41].

The power-law model was found to be fairly fitted (R^2^ > 0.8) to both upward and downward flow curves of the double emulsions, except the upward curve of the sample named 5%P-3%N and the downward curves of samples named 3%P@3%N and 5%P@3%N. The power-law model was fitted to understand the flow behavior in detail, and the parameters of the model are displayed in Table 3. The emulsions, which fairly fitted the equation, had *n* values less than unity and thus demonstrated a shear-thinning (pseudoplastic) behavior. The lower the value of *n*, the higher the degree of shear thinning [42]. The stable shear rheological parameters of the double emulsion were found to be dependent on the PGPR and NCC concentrations. Regarding the flow behavior index (*n*), indications of shear-thinning and non-Newtonian flow behavior were observed in formulations with a lower concentration of NCC.

**Figure 4:**
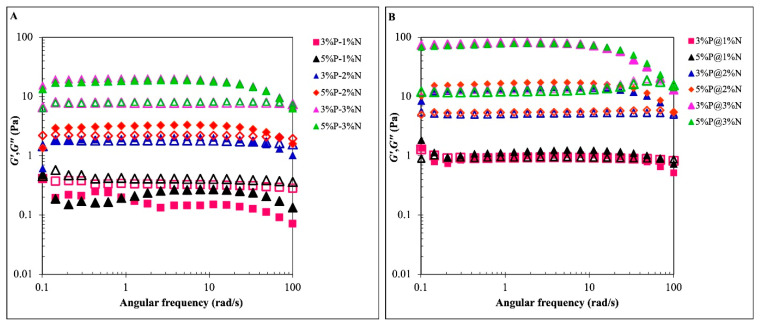
Dynamic rheology of BPE-loaded double Pickering emulsions stabilized by various PGPR and NCC concentrations and prepared with different phase volumetric ratios of W_1_/O in W_2_ at 2:8 (**A**) and 3:7 (**B**). Note: it is indicated as the storage modulus, G′ (closed symbols), and loss modulus, G″ (open symbols), as a function of angular frequency.

The measurements from dynamic oscillations supported the conclusions drawn from the shear behavior. For the different concentrations of PGPR and NCC within the inner-to-outer phase ratios of 2:8 and 3:7 of the BPE-loaded double emulsion, the dynamic mechanical spectra characterizing the viscoelastic behavior are shown in Figure 4A,B. The storage modulus (G′) and loss modulus (G″), which are oscillatory rheological measures, show the relative stiffness or weakness of the flocculated emulsion system [15]. The G′ in the formulation with 1% NCC and 3% P-2%N was less than or equal to the G″, as shown in Figure 4A, and the trend was consistent across the entire frequency range. Under oscillatory shear conditions, the aqueous medium predominated the deformation because the NCC content was insufficient for interaction. As a result, this emulsion tended to flow easily and showed a liquidlike behavior, which indicated that it was unstable in storage [34,43]. Furthermore, G″ values were still higher than G′ in certain samples (3%P-1%N and 5%P-1%N) despite having a higher PGPR, indicating liquidlike properties. Thus, the PGPR concentration had little or no effect.

In the double emulsions containing NCC concentrations higher than 2% *w*/*v*, except for 3% PGPR, the G′ values were significantly higher than G″ throughout the tested frequency range (Figure 4A,B). It revealed that elastic, solidlike, or gel-like characteristics predominated, which was also supported by frequency sweep results. This can be attributed to the fact that the nanocellulose particles themselves were at high concentrations, where the nanocellulose forms a gel network within a continuous phase without any dispersed oil droplets. The stiffness increased at higher concentration of NCC as the more numerous free NCC particles are available in continuous phase [44]. These W_1_/O/W_2_ emulsions also had a high viscosity (*η*) as well as favorable shear-thinning properties (Figure 3A,B). Hence, such bulk elasticity is related to the close packing, which has often been described in the presence of a higher concentration of biopolymers in solutions [27].

However, dispersed oil droplets may also contribute to improving the storage modulus due to greater interactions between NCC nanoparticles on distinct oil droplets but do not necessarily form a close packing for emulsion elasticity [27,44]. Collectively, the dynamic viscosity also demonstrated the level of resistance of the droplets to shear-induced structural disintegration.

### 3.3. Encapsulation Efficiency and Storage Stability

**Figure 5:**
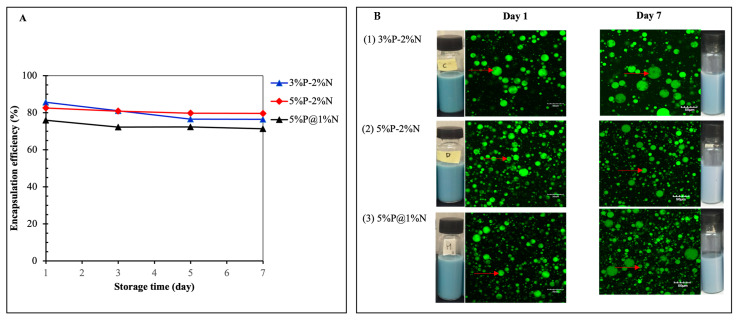
Encapsulation efficiency (**A**) and confocal laser scanning micrographs and visual images (**B**) of the optimized BPE-loaded double Pickering emulsions during storage at room temperature (25 ± 2 °C) for 7 days. Red arrows mark the incorporated inner aqueous phase.

The results from Section 3.1 and Section 3.2 indicate that 3% P-2%N, 5% P-2%N, and 5% P@1%N were the stable emulsion formulation compositions in terms of their emulsion creaming, color, particle size distribution, and ζ-potential. Consequently, these formulations were selected for a further investigation of their storage stability, microstructure, and encapsulation efficiency. The stability of anthocyanin in selected samples was determined and plotted as a time function. The total anthocyanin content was analyzed and observed throughout 7 days of storage under room conditions. The findings revealed that longer storage conditions led to a decrease in the overall anthocyanin content of all samples. The concentration of PGPR in the oil phase and NCC in the outer aqueous phase may be associated with satisfactory stability results. After seven days of storage at 25 °C, the anthocyanin concentration in the internal aqueous phase of the double emulsion decreased slightly (Figure 5A). In samples 3%P-2%N, 5%P-2%N, and 5%P@1%N, the total anthocyanin content decreased from 89.83% to 76.49%, 89.40% to 79.65, and 86.63% to 71.40%, respectively. This could be attributed to the overflow of anthocyanins from W_1_ to W_2_ as a result of the condensation of inner W_1_ water droplets driven by an osmotic pressure gradient [10]. In contrast to double emulsions with 1% NCC concentration at an outer-phase ratio of 3:7, emulsions with 2% NCC concentration and an outer-phase ratio of 2:8 demonstrated a high encapsulation efficiency and stability. Nevertheless, no significant difference in encapsulation efficiency was observed when the PGPR concentration increased from 3.0% to 5.0%, similar to an earlier study by Lin et al. [10]. 

The results of encapsulation efficiency were further evidenced by CLSM, in which the intermediate oil phase appeared green and the inner water droplets were dark inside the green oil droplets (Figure 5B, Day 1). The emulsion system was able to incorporate anthocyanin into its inner phase and successfully maintain its stability. After 7 days of storage, an increase in the size of the W1/O droplets was visible through the emulsion network (Figure 5B, Day 7). This could be due to water diffusion from the external to the internal aqueous phase, which is induced by the difference in osmotic pressure between the two aqueous phases [45].

## 4. Conclusions

In the current investigation, a double-emulsion system stabilized by PGPR and NCC was used successfully to encapsulate BPE at a neutral pH. A double emulsion stabilized with an appropriate concentration of hydrophobic (PGPR) and hydrophilic (NCC) stabilizers exhibited a vivid green color and showed an ability for incorporating anthocyanin with a higher stability. A microstructure examination after 7 days of storage showed that the majority of oil droplets encapsulated the inner aqueous phase (BPE at pH 7). Even though formulations with 2% and 3% NCC might potentially be employed for food purposes, the formulation with 5% PGPR, 2% NCC, and a 2:8 W_1_/O-to-W_2_ ratio was chosen as the optimum formulation due to its high loading capacity, favorable rheological characteristics, and increased color stability. In this context, double emulsions were investigated to serve as delivery mediums that were primarily designed for the encapsulation of BPE. For the implementation of the produced double emulsions in enriched food products, additional research is required to assess their release characteristics as well as their effects on the physicochemical and sensory attributes of food products.

## Figures and Tables

**Table 1:** Composition of various treatments employed for the production of oil-in-water-in-oil (W_1_/O/W_2_) emulsions with a constant ratio of inner water phases (W_1_) in oil at 3:7.

Sample Code	Treatments	Phase Volume (W_1_:O) in W_2_	PGPR (%)	NCC (%)
A	3%P-1%N	2:8	3	1
B	5%P-1%N	2:8	5	2
C	3%P-2%N	2:8	3	3
D	5%P-2%N	2:8	5	1
E	3%P-3%N	2:8	3	2
F	5%P-3%N	2:8	5	3
G	3%P@1%N	3:7	3	1
H	5%P@1%N	3:7	5	2
I	3%P@2%N	3:7	3	3
J	5%P@2%N	3:7	5	1
K	3%P@3%N	3:7	3	2
L	5%P@3%N	3:7	5	3

(-) indicates W_1_/O in W_2_ (2:8) and (@) indicates W_1_/O in W_2_ (3:7) with varying percentages of PGPR and NCC, except wherever mentioned.

**Table 2:** Color (L*, a*, b*) and droplet diameter D [4,3] of BPE-loaded double Pickering emulsions stabilized by various PGPR and NCC concentrations and prepared with different phase volumetric ratios of W_1_/O in W_2_.

Sample	L*	a*	b*	D [3,2] (μm)
3%P-1%N	49.0 ± 1.2 ^ab^	−10.8 ± 0.2 ^a^	−10.1 ± 0.8 ^a^	12.3 ± 0.8 ^bc^
5%P-1%N	49.7 ± 2.1 ^abcd^	−10.5 ± 0.2 ^bcd^	−10.3 ± 0.8 ^a^	11.0 ± 0.9 ^abc^
3%P-2%N	48.8 ± 0.2 ^a^	−10.6 ± 0.1 ^abc^	−10.4 ± 0.5 ^a^	12.4 ± 0.7 ^c^
5%P-2%N	50.5 ± 0.6 ^abcd^	−10.2 ± −0.1 ^de^	−10.1 ± 0.6 ^a^	9.9 ± 1.1 ^a^
3%P-3%N	50.4 ± 1.1 ^abcd^	−10.4 ± 0.1 ^cde^	−9.4 ± 1.0 ^a^	12.4 ± 0.6 ^c^
5%P-3%N	51.8 ± 1.5 ^d^	−10.2 ± 0.1 ^e^	−9.4 ± 1.0 ^a^	9.9 ± 0.9 ^a^
3%P@1%N	50.3 ± 0.6 ^abcd^	−10.8 ± 0.1 ^a^	−10.0 ± 0.3 ^a^	10.5 ± 0.5 ^ab^
5%P@1%N	50.3 ± 2.2 ^abcd^	−10.4 ± 0.2 ^cd^	−10.3 ± 0.6 ^a^	10.7 ± 1.2 ^abc^
3%P@2%N	49.1 ± 0.6 ^abc^	−10.8 ± 0.2 ^ab^	−9.9 ± 0.6 ^a^	12.6 ± 0.9 ^bc^
5%P@2%N	50.1 ± 0.9 ^abcd^	−10.6 ± 0.1 ^abc^	−10.2 ± 0.5 ^a^	10.4 ± 0.2 ^abc^
3%P@3%N	51.3 ± 0.1 ^cd^	−10.4 ± 0.2 ^cd^	−9.2 ± 0.3 ^a^	11.3 ± 0.8 ^abc^
5%P@3%N	51.6 ± 1.0 ^bcd^	−10.2 ± 0.1 ^de^	−9.3 ± 0.5 ^a^	10.4 ± 1.1 ^a^

Different lowercase letters (a–e) in the same column result in a significant (*p* ≤ 0.05) difference concerning different formulations.

**Table 3:** Power-law model parameters of BPE-loaded double Pickering emulsions stabilized by various PGPR and NCC concentrations and prepared with different phase volumetric ratios of W_1_/O in W_2_.

W_1_/O in W_2_ (*w*/*v*) (2:8)				W_1_/O in W_2_ (*w*/*v*) (3:7)			
Sample	*K* (Pa·s^n^)	*n* (-)	R^2^	Sample	*K* (Pa·s^n^)	*n* (-)	R^2^
3%P-1%N	0.033	0.351	0.988	3%P@1%N	0.183	0.341	0.976
5%P-1%N	0.063	0.333	0.981	5%P@1%N	0.348	0.327	0.958
3%P-2%N	0.217	0.533	0.958	3%P@2%N	2.968	0.427	0.846
5%P-2%N	0.339	0.547	0.957	5%P@2%N	3.069	0.353	0.829
3%P-3%N	2.041	0.685	0.837	3%P@3%N ^a^	-	-	-
5%P-3%N ^a^	-	-	-	5%P@3%N ^a^	-	-	

^a^ Power-law equation is not fitted to the curves of flow.

## Data Availability

Data is contained within the article or Supplementary Materials.

## Supplementary Materials

The following supporting information can be downloaded at: https://www.mdpi.com/article/10.3390/foods12224173/s1. Figure S1: HPLC spectrum of butterfly pea petal extract (UAE method) (Peak 1 RT 24.544 min, Peak area: 208.109); Figure S2: Particle size distribution of BPE-loaded double Pickering emulsions stabilized by various PGPR and NCC concentrations and prepared with different phase volumetric ratios of W_1_/O in W_2_ at 2:8 (**A**) and 3:7 (**B**).
