# Supplementary material for: Anthocyanin-Rich Butterfly Pea Petal Extract Loaded Double Pickering Emulsion Containing Nanocrystalline Cellulose: Physicochemical Properties, Stability, and Rheology
Source: Foods. 2023 Nov 19;12(22):4173. doi: 10.3390/foods12224173 (PMC10671032; doi:10.3390/foods12224173)
Supplement: Supplementary file 1 [file foods-12-04173-s001.zip › foods-2712283-supplementary.pdf]

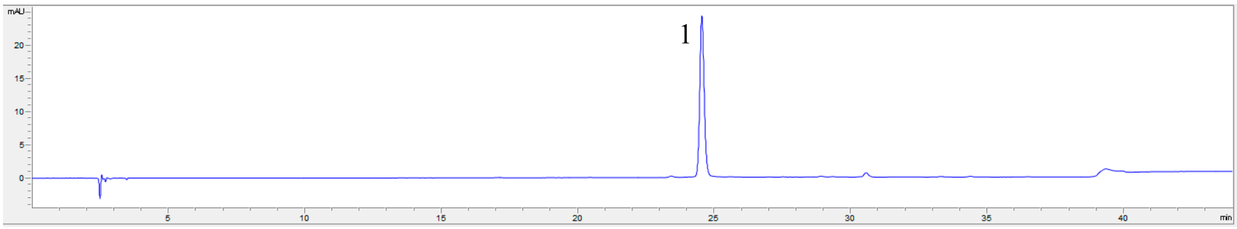

Figure S1 HPLC spectrum of butterfly pea petal extract (UAE method) (Peak 1 RT 24.544 min, Peak area: 208.109)

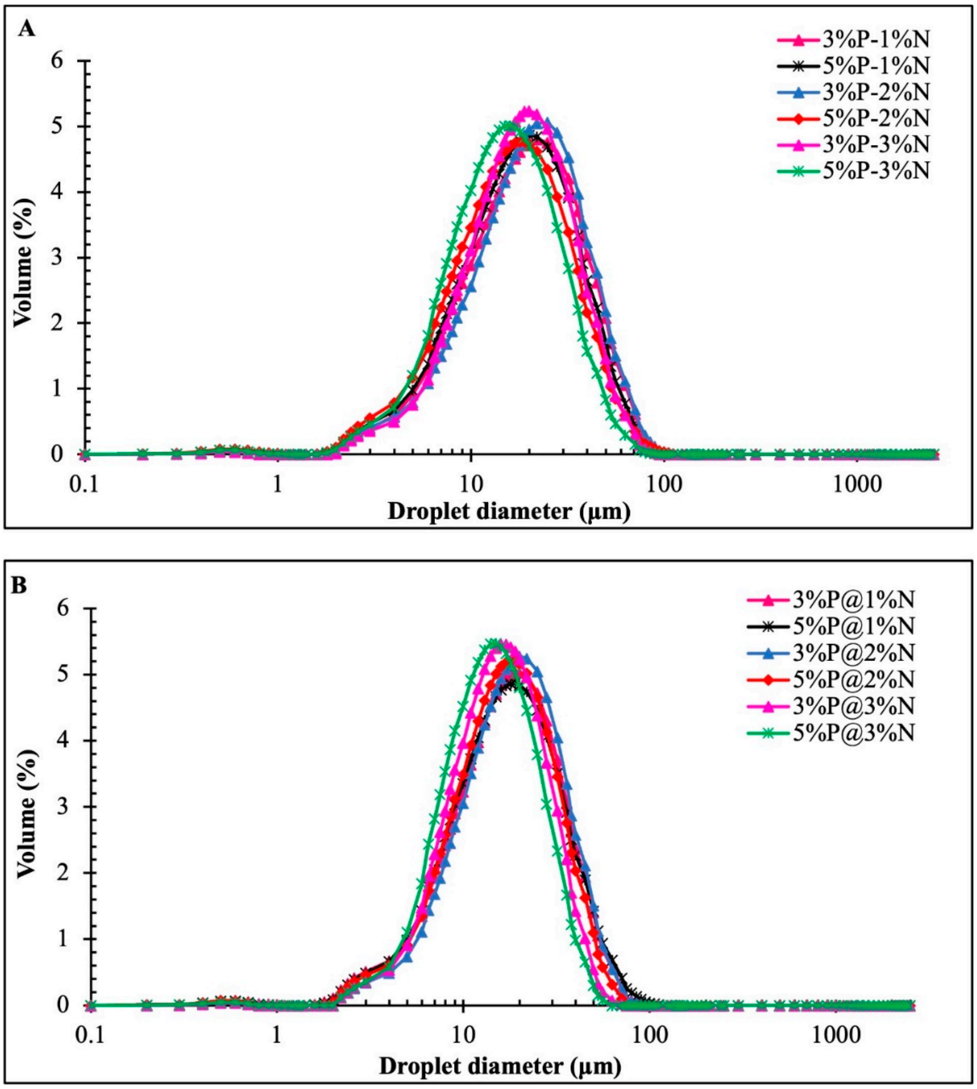

Figure S2 Particle size distribution of BPE-loaded double Pickering emulsions stabilized by various PGPR and NCC concentrations and prepared with different phase volumetric ratios of W_1/O in W_2 at 2:8 (A) and 3:7 (B)
